# Pocket Parent-Child Interaction Therapy (PCIT) Online for Young Children With Disruptive Behaviors: Open Trial

**DOI:** 10.2196/69887

**Published:** 2025-08-08

**Authors:** Jason F Jent, Megan Golson, Abigail Peskin, William A Rothenberg, Hanan Salem, Allison Weinstein, Eileen Davis, Meaghan Parlade, Jocelyn Stokes, Tasha Brown, Michelle Berkovits, Dainelys Garcia

**Affiliations:** 1 Department of Pediatrics, University of Miami Miller School of Medicine, 3047E 12th Ave, Miami, FL, 33136, United States, 1 3052436857; 2 West Virginia University Hospitals, Morgantown, WV, United States; 3 Private Practice, New York, NY, United States

**Keywords:** Parent-Child Interaction Therapy, PCIT, behavioral parent training, mHealth intervention, child disruptive behavior, online parenting program, RE-AIM framework, intervention engagement

## Abstract

**Background:**

Parent-Child Interaction Therapy (PCIT) is an evidence-based treatment for child disruptive behavior problems, but access barriers historically limit its reach.

**Objective:**

This study examined the reach, effectiveness, adoption, and implementation of Pocket PCIT Online, a self-directed web-based adaptation of PCIT.

**Methods:**

In an open trial, 1480 caregivers accessed the free 4-week Pocket PCIT Online intervention. Measures of child behavior, parenting stress, and family conflict were collected pre- and postintervention. Reach, effectiveness, adoption, and implementation were assessed using an implementation science framework.

**Results:**

Significant improvements were observed across all outcome measures for intervention completers (n=204). Caregivers reported increased positive child behaviors (Cohen’s *d*=0.87) and decreased parenting distress (Cohen’s *d*=−0.3) following completion of Pocket PCIT Online. Of note, approximately 35.8% (73/204) of caregivers reported clinically significant improvements in their children’s frequency of disruptive behaviors. However, only 16.5% (204/1234) of participants completed postintervention measures. Caregivers completed Pocket PCIT Online at a significantly higher rate prior to the COVID-19 national emergency (21/74, 28%) than during or after the onset of COVID-19 (183/1158, 15.8%).

**Conclusions:**

While Pocket PCIT Online demonstrates potential as a low-cost, accessible, and scalable public health intervention for child disruptive behaviors, strategies to enhance retention and broaden reach to historically underserved populations are needed.

## Introduction

### Early Childhood Disruptive Behavior Problems and the Role of Behavioral Parent Training

Though mild disruptive behaviors such as aggression, tantrums, and noncompliance are common in early childhood, clinically significant levels of these behaviors can increase the risk of impairment and child and family dysfunction [1]. In 2011, the estimated prevalence of significant disruptive behavior problems among children aged 3-17 years in the United States was 3.5% [2]. By 2016, the prevalence of disruptive behavior problems of children aged 3-17 years in a separate study was estimated to be 7.4% [3]. If left untreated, disruptive behavior problems are associated with several suboptimal long-term outcomes, including future risk for academic challenges, conduct problems, adult psychiatric disorders, encounters with criminal justice, substance abuse disorders, occupational challenges, and housing instability [1,4-6].

To mitigate child disruptive behavior problems, several professional organizations, including the American Academy of Child and Adolescent Psychiatry and the American Academy of Pediatrics, recommend behavioral parent training (BPT) as the primary intervention [7,8]. BPT is widely regarded as a well-established and effective intervention for childhood disruptive behavior problems [9,10]. BPT programs, such as Incredible Years, Parent Management Training–Oregon Model, Triple P Positive Parenting, and Parent-Child Interaction Therapy (PCIT), share a theoretical basis in family systems theory, social learning theory, and operant conditioning [11-15]. These programs emphasize key strategies such as differential caregiver attention, effective delivery of commands, and consistent consequences for noncompliance, which are taught to caregivers using didactic instruction, modeling, and live coaching of caregivers with their children [16].

BPT programs have a strong track record of effectively reducing disruptive behaviors in children. Moderate improvements in child behavior have been shown even for families who complete a portion of the BPT programs [17]. However, greater levels of engagement (eg, attendance and homework completion) are associated with greater improvements across child and family outcomes, with families who complete treatment demonstrating the greatest benefit [17,18]. In addition to reductions in disruptive behavior problems, BPT has been shown to improve other family-level outcomes, including parenting stress, parenting self-efficacy, family conflict, and the use of effective parenting strategies [19-21].

### Access and Engagement

Despite the effectiveness of BPT programs, access to these interventions remains a significant challenge. Only about 10% of children in need of mental health services receive treatment [22]. Challenges related to access are compounded by high attrition rates, with more than 50% of those who initiate services discontinuing before completion [23]. A meta-analysis on caregiver engagement in BPT reported a similar attrition rate of 51%, with even higher rates reported among ethnically minoritized families and families of lower socioeconomic status [24,25]. Multiple barriers contribute to limited access and high attrition, including knowledge of available services, financial barriers, lack or shortages of trained providers, and lengthy waitlists [26-29]. Additional logistical barriers, such as childcare, transportation, scheduling conflicts, and competing family stressors, further hinder engagement [19,30,31].

### The Potential of Technology: Web-Based Parenting Programs

The potential of technology to address BPT access and engagement has gained significant research attention over the past decade. Several meta-analyses have shown that mobile health (mHealth) BPT effectively reduces child behavior problems, with effect sizes ranging from small to moderate [32-34]. Additionally, these interventions have been shown to positively impact caregiver use of positive parenting skills, parenting self-efficacy, parenting stress, and parenting knowledge [33,35,36]. Further, a qualitative study revealed that 70% of caregivers who discontinued BPT expressed interest in hybrid services, such as internet-facilitated BPT, to reduce barriers [37].

Despite the benefits of mHealth BPT programs at the child and family levels, there is a lack of consensus regarding the optimal delivery format (eg, benefits of additional consultation) and the specific populations for whom mHealth BPT may be most beneficial, given variations in effectiveness based on socioeconomic factors (eg, caregivers with higher education and income levels reporting larger reductions in child disruptive behaviors) [38]. No differences in behavioral outcomes have been found across mHealth BPT programs serving racially minoritized families compared to programs serving predominantly White families [39].

Maintaining engagement with mHealth BPT remains a challenge [40]. Research examining the sociodemographic characteristics of families who have accessed mHealth BPT programs has yielded mixed findings regarding engagement. Notably, caregiver race, ethnicity, and income level generally do not predict program completion or retention [41]. However, caregiver factors such as higher parenting self-efficacy, greater internet usage, and fewer depressive symptoms at baseline are associated with greater engagement in mHealth BPT [41,42]. Additionally, lower ratings of child behavior problems at baseline have been associated with higher engagement and completion rates [43]. Qualitatively, caregivers report that time constraints, technical difficulties (eg, internet connectivity), and registration requirements (eg, providing an email and pretest measures) contribute to discontinuing mHealth BPT programs [44].

### Advancing and Evaluating mHealth BPT

Despite strong evidence for mHealth BPT, several critical challenges remain unaddressed in this field. While initial findings highlight the efficacy of these programs, challenges include the identification of optimal program delivery formats, the pinpointing of specific populations who may derive the greatest benefit from these interventions, and the persistent difficulty of sustaining caregiver engagement with online content. To more fully understand the broader impact and potential of mHealth BPT programs, it is crucial to examine them through a more comprehensive implementation science lens. The RE-AIM (Reach, Effectiveness, Adoption, Implementation, and Maintenance) framework, a tool used to evaluate public health interventions and programs, offers a valuable approach for this purpose [45].

Very few mHealth BPT programs have been examined through implementation science frameworks [46]. Aspects of the RE-AIM framework provide researchers and practitioners with a more holistic view of mHealth BPT, addressing not just their efficacy but also their practical implementation. This approach could inform strategies to overcome engagement and retention challenges, ultimately enhancing the population-level scaling of mHealth BPT programs. Measurement of the *Reach* domain provides important information regarding the extent to which mHealth BPT programs are reaching their intended audience. Given mHealth BPT engagement challenges, analyzing *Reach* could reveal whether certain sociodemographic characteristics of families are underrepresented relative to the general population and could inform future strategies to improve access. While existing literature supports the effectiveness of mHealth BPT, the RE-AIM approach encourages a more nuanced examination of *effectiveness,* including assessing who the intervention works best for and the potential heterogeneity of outcomes across different subgroups. Evaluating *adoption* rates among caregivers could illuminate facilitators and barriers to engagement in mHealth BPT programs, which is crucial for scaling up effective interventions. Given retention challenges in mHealth BPT interventions, examining *implementation* factors such as caregiver adherence to mHealth BPT intervention models (eg, homework completion) may provide valuable insights into factors affecting participant engagement. Finally, when feasible, assessment of long-term outcomes and program sustainability or *maintenance* is critical for understanding the lasting impact of mHealth BPT programs. Evaluation of these factors through aspects of the RE-AIM framework is essential for bridging the gap between the proven effectiveness of mHealth BPT interventions and their successful implementation in diverse real-world contexts.

### Pocket PCIT Online

Given the need for a more holistic evaluation of mHealth BPT, this study aimed to examine an mHealth version of PCIT through the aspects of the RE-AIM framework lens. Building on the success of a PCIT-related multimedia book [47], the content was adapted and expanded into a web-based platform. Pocket PCIT Online is a brief, self-directed web-based adaptation of Parent-Child Interaction Therapy, developed to increase accessibility of evidence-based parenting strategies [14,48-50]. Building on the Pocket PCIT eBook’s success [47], this 4-week intervention mirrors PCIT’s Child-Directed Interaction (CDI) and Parent-Directed Interaction (PDI) phases. The CDI Phase includes 6 online modules, and the PDI Phase includes 11 online modules. The expected time to read module content, watch skill explanations and demonstration videos, and engage in interactive activities (eg, Labeled Praise Mixer and Knowledge Checks) ranges from 5 to 15 minutes per module. Modules offer multimedia content teaching positive parenting skills, effective discipline, and applied PCIT principles [51-54]. Key features include on-demand access, multimedia education on PCIT skills, flexible navigation of parenting topics, daily skill practice guidance, and written content at a 7th-grade reading level. At the end of each CDI Phase module, caregivers were assigned a daily special time practice with their child to practice strategic attention and selective ignoring. Once caregivers believed that they were demonstrating skill proficiency in CDI Skills (10 labeled praises, 10 behavior descriptions, 10 reflections, and 3 or fewer commands, questions, and criticisms within a 5-minute period), it was recommended that they proceed to the PDI Phase modules and add daily PDI practice with their children, which emphasizes delivering effective commands and correct follow-through. Unlike traditional PCIT, Pocket PCIT Online does not include guided practice or coaching and uses a return-to-chair procedure instead of a time-out room as a backup to the time-out chair [55]. The website content is only presented in English, with efforts made to incorporate diverse family representation, although the clinicians featured are primarily non-Hispanic White.

By adapting PCIT to a mobile platform (Pocket PCIT Online) and systematically evaluating it using *reach*, *effectiveness*, *adoption*, and *implementation*, we sought to provide a comprehensive understanding of its potential for widespread implementation and impact. *Maintenance* was not evaluated in this initial open trial due to resource limitations. Specifically, we sought to examine the following research questions.

- **Reach:** What are the sociodemographic characteristics of families who access Pocket PCIT Online? To what extent does Pocket PCIT Online reach its intended target population? Are there significant differences between those who access the program and the broader population in need?
- **Effectiveness:** What is the impact of Pocket PCIT Online completion on family outcomes? To what degree do caregiver factors and sociodemographic characteristics predict family outcomes following intervention?
- **Adoption:** What are the engagement rates for families who begin Pocket PCIT Online? What factors predict higher levels of homework completion related to the Pocket PCIT intervention? What caregiver factors and sociodemographic characteristics are associated with program retention?
- **Implementation:** To what degree do caregivers adhere to the prescribed Pocket PCIT Online assigned homework? What are the ongoing costs associated with maintaining the Pocket PCIT Online platform?

This multifaceted investigation will not only advance our understanding of the complex interplay between Pocket PCIT Online characteristics and outcomes, but it will also inform the future design of more targeted and efficient mHealth BPT that are personalized to the needs and preferences of diverse families.

## Methods

### Participants

Participating caregivers (N=1480) enrolled in the Pocket PCIT Online open trial from July 1, 2019, to June 30, 2024. Pocket PCIT Online was advertised on a national parenting website related to PCIT [56] and to English-speaking families at a local PCIT clinic when they were being placed on a waitlist for services. Pocket PCIT Online content was freely available to any caregivers with children ages 2‐7 years who completed informed consent for this study. Demographic information was collected through an online database management system, Research Electronic Data Capture (REDCap) [57], wherein caregivers self-selected responses. For additional demographic characteristics, see Table 1. Study inclusion criteria consisted of (1) being the caregiver of a child between the ages of 2 and 7 years, (2) being fluent in English (as text and videos were only available in English for the pilot trial), and (3) expressing caregiver concerns about their children’s disruptive behaviors.

**Table 1.** Demographic information for Pocket PCIT^a^ Online participants.

Characteristic	Values
Accessed Pocket PCIT Online and completed informed consent (n=1480), n (%)	1480 (100)
Accessed Pocket PCIT Online but did not complete preintervention measures (n=1480), n (%)	246 (16.6)
Completed preintervention measures (n=1480), n (%)	1234 (83.4)
Completed any Pocket PCIT homework (n=1234), n (%)	393 (31.8)
Completed Pocket PCIT Online (n=1234), n (%)	204 (16.5)
Child gender (n=1234), n (%)	
Male	818 (66.3)
Female	416 (33.7)
Caregiver age (years; n=1234), n (%)	
18‐24	23 (1.9)
25‐44	1048 (84.9)
45‐64	160 (13)
65 or older	3 (0.2)
Caregiver education (n=1234), n (%)	
<Bachelor’s degree	285 (23.1)
Bachelor’s degree or higher	949 (76.9)
US median household income range (n=1234), n (%)	
Below the US household median income	429 (34.8)
At or above the US household median income of US $74,580	805 (65.2)
Child race (n=1234), n (%)	
American Indian or Alaska Native	
Hispanic	10 (0.8)
Non-Hispanic	14 (1.1)
Asian	
Hispanic	6 (0.5)
Non-Hispanic	90 (7.3)
Black or African American	
Hispanic	12 (1.0)
Non-Hispanic	71 (5.8)
Native Hawaiian or Other Pacific Islander	
Hispanic	4 (0.3)
Non-Hispanic	9 (0.7)
White	
Hispanic	193 (15.6)
Non-Hispanic	825 (66.9)
Total	
Hispanic	225 (18.2)
Non-Hispanic	1009 (81.8)
Child age (years), mean (SD)	4.73 (1.58)
PSI-SF^b^ Parental Distress preintervention raw score, mean (SD)	33.30 (9.16)
WACB-P^c^ preintervention total score, mean (SD)	32.53 (6.68)

a PCIT: Parent-Child Interaction Therapy.

b PSI-SF: Parenting Stress Index – Short Form.

c WACB-P: Weekly Assessment of Child Behavior – Positive.

### Ethical Considerations

Institutional review board approval was obtained from the University of Miami (study #20161163), and all participants who agreed to be in the study signed an informed consent. All study procedures were conducted in accordance with the ethical standards of the institutional review board. All data were collected and managed through a secure online data capture system, REDCap. Participant protected health information was deidentified. Participants did not receive any form of compensation for participation and could opt out of the study at any time, but could still access the content of Pocket PCIT Online.

### Procedures

Caregivers completed preintervention measures, including sociodemographic information and caregiver reports of parenting stress, family conflict, and their children’s disruptive behaviors. Caregivers were then provided with instructions on how to access and use Pocket PCIT Online. Caregivers could access and complete Pocket PCIT Online modules at any time that they preferred during the 4-week period. Caregivers were provided the lead investigator’s contact information if they experienced technical difficulties accessing the website. Following preassessments, caregivers received a weekly email reminder (for 3 weeks) to practice their Pocket PCIT Online skills during daily special time practice with their child. Each week, caregivers reported the number of days they practiced these skills with their child during special time. Following 4 weeks of Pocket PCIT Online use, caregivers again rated their parenting stress, level of family conflict, and their children’s disruptive behaviors, and they completed a satisfaction questionnaire about the intervention.

Of the 1480 families enrolled, 1234 (83.4%) completed a preintervention assessment. Of those who completed the initial assessment, 204 (16.5%) caregivers also completed a postintervention assessment. Beyond caregiver reports and characteristics, Pocket PCIT Online website utilization and costs were tracked during the study period.

### Measures

#### Reach Measures

##### Sociodemographic Questionnaire

Information about the family (eg, caregiver role, gender, race, ethnicity, education level, income, and language) was collected. Participants were coded based on their intervention timeline relative to the COVID-19 national emergency declaration (March 13, 2020). Families expected to complete their 4-week intervention after this date were coded as 1 (COVID or after), while those expected to complete Pocket PCIT Online before this date were coded as 0 (pre-COVID).

##### Pocket PCIT Online Access

Intervention access was measured as the percentage of caregivers who completed preintervention measures relative to the total number of caregivers who registered to use the Pocket PCIT Online website. A link to access Pocket PCIT Online was provided to participants when they completed the informed consent.

### Effectiveness Measures

#### Weekly Assessment of Child Behavior – Positive

The WACB-P (Weekly Assessment of Child Behavior – Positive) measured caregiver reports of the frequency that their children engaged in positive child behaviors and the number of types of behaviors they would like to change in their child [58]. This instrument is a brief, 9-item caregiver-report measure designed to assess changes in children’s positive behaviors on a weekly basis. Caregivers rate each item on a Likert scale, ranging from 1 to 7, reflecting the frequency of these behaviors over the past week. Higher scores indicate a greater frequency of positive child behaviors; total scores below 37 are considered clinically significant [58]. Caregivers also rate the number of the 9 listed behaviors that they would like to change in their child (ranging from 0 to 9). Higher scores represent a greater number of total behavior problems. The WACB-P has previously demonstrated strong convergent validity with the Eyberg Child Behavior Inventory [59], the standard measure for intensity and number of child disruptive behaviors in PCIT specifically [58]. The WACB-P within this study demonstrated good internal consistency at baseline (Cronbach α=0.84) and good test-retest reliability (*r*=0.63, *P*<.001).

#### Parenting Stress Index – Short Form: Fourth Edition (PSI-SF-4) Parental Distress

This subscale was used to assess caregivers’ stress levels related to their parenting role and is part of the broader PSI-SF-4, a widely used and validated measure in child and family research [60]. The Parental Distress subscale consists of 12 items that evaluate the distress a caregiver experiences due to personal factors directly related to parenting. Caregivers respond to each item on a 5-point Likert scale. Higher scores indicate higher levels of parenting-related distress. Within the current sample, the subscale demonstrated good internal consistency at baseline (Cronbach α=0.88) and test-retest reliability (*r*=0.69, *P*<.001).

#### Family Conflict Scale

Family conflict was measured using the 5-item family conflict subscale of Bloom’s Family Processes Scale [61]. Participants rated the extent to which they agreed that a statement reflected their family life over the past month using a 5-point response scale, with higher scores reflecting higher family conflict over the past month. This subscale was found to have adequate validity and internal reliability in previous studies [61]. In this study, internal consistency at baseline was adequate (Cronbach α=0.73).

### Adoption Measures

#### Any Pocket PCIT Online Engagement

Caregivers were rated as engaging in Pocket PCIT Online if they reported completion of homework practice at least once during the intervention (1=engaged, 0=did not engage). Examination of who initially engages in homework completion and who does not may provide vital clues for how to refine the intervention to increase the number of caregivers who begin to practice the learned skills with their own child.

#### Pocket PCIT Online Retention

Caregivers who reported completing a minimum of one CDI or PDI homework assignment and completed pre- and postintervention measures were listed as study completers (1=completed; 0=did not complete).

#### Adapted Therapy Attitude Inventory (TAI)

Caregiver attitudes toward Pocket PCIT Online were assessed using an adapted version of the TAI [62]. The original TAI is a 10-item self-report questionnaire that measures caregiver satisfaction with parenting programs. It uses a 5-point Likert scale, with higher scores indicating greater satisfaction or agreement with the statement. For this study, the TAI was modified to capture satisfaction specific to the Pocket PCIT Online experience. The adapted TAI within the current sample demonstrated excellent internal consistency (Cronbach α=0.90). For this study, individual item descriptive statistics were examined to explore specific elements of Pocket PCIT Online which may impact its adoption.

### Pocket PCIT Web Page and YouTube Metrics

Due to the web platform’s tracking limitations, individual user metrics for Pocket PCIT Online were not available. Instead, to gauge overall engagement, we relied on aggregate data. Specifically, we monitored total web page views for Pocket PCIT throughout the study period, providing a general indication of website utilization regardless of study measure completion. Additionally, we tracked the total watch time of embedded Pocket PCIT YouTube videos on the website during the study timeframe. These aggregate measures, while not user-specific, offered insights into the general Pocket PCIT usage.

### Implementation Measures

#### CDI and PDI Homework Completion

The homework completion measure used in this study assessed caregivers’ reported adherence to daily CDI and PDI skills practice, as recommended by the PCIT protocol [14]. During the 4-week mHealth intervention, caregivers were asked weekly to report the frequency with which they practiced CDI and PDI skills [63].

#### Pocket PCIT Online Ongoing Operational Costs

Understanding the implementation cost is essential for organizations considering adopting and sustaining the program. Therefore, we measured the cost of hosting Pocket PCIT Online over the study period. The cost of the website platform and domain name from 2019 to 2024 was US $1895.

### Data Analytic Plan

Descriptive statistics, correlation, chi-square, and *t* test analyses were completed in SPSS 29.0 (IBM Corporation). All regression analyses were conducted within Mplus 8.10 (Muthén & Muthén). Power analyses for the Pocket PCIT Online intervention were based on a similar self-directed, paper-based bibliotherapy version of PCIT, PCIT-Anticipatory Guidance [55]. Based on this prior work, power analyses indicated that a minimum of 61 participants would be needed to detect the expected medium effect size (*d*=0.51) with 80% power and an α set at .05. To account for the notoriously high attrition rates in digital interventions (with an average retention rate of only 6% across apps [64]), we established a recruitment target of a minimum of 1000 participants to ensure adequate statistical power for our analyses.

Reach analyses included descriptive statistics, which were calculated for all sociodemographic variables. Caregiver sociodemographic characteristics in the Pocket PCIT sample were descriptively compared to national estimates from the U.S. Census [65-67]. Effectiveness analyses included paired sample *t* tests that were conducted to examine pre-post changes in child frequency of positive behaviors, parental distress, and family conflict, and the magnitude of changes. Bivariate correlations were conducted to examine the extent that different sociodemographic characteristics and caregiver factors impacted changes in outcome variables. Any variable that was correlated with postoutcome scores was included in multiple linear regression analyses. To examine predictors of postintervention family outcomes, we conducted multiple regression analyses. Within each regression analysis, prelevels of the postmeasure were held constant to examine the extent that additional independent variables, including caregiver factors and sociodemographic characteristics, predicted family outcomes. Specifically, regression analyses were conducted to examine the extent that the independent variables including sociodemographic characteristics (categorical variables dummy coded for the analysis; ie, child race, child age, and caregiver education), preintervention family measures (pre–WACB-P Frequency, pre–PSI-SF Parental Distress, and pre–Family Conflict), and CDI homework completion rate predicted the outcome variables (post–WACB-P, post–PSI-SF Parental Distress, and post–Family Conflict). All predictor variables were entered simultaneously into one model or block of the linear regression model. We used robust maximum likelihood estimation (MLR) to account for potential nonnormality in the data. Missing data were handled using full information maximum likelihood (FIML), as indicated by the TYPE=MISSING command. This approach allowed for the inclusion of all available data, potentially reducing bias and increasing statistical power compared to listwise deletion [68].

For measuring adoption, within SPSS 29.0 (IBM Corporation), chi-square analyses were calculated to determine if there were any sociodemographic differences or COVID-19–related factors between caregivers who completed Pocket PCIT Online and those lost to follow-up. Preliminary correlations were conducted to examine the extent that dummy-coded sociodemographic variables and caregiver predictor variables were correlated with any engagement in Pocket PCIT Online and Pocket PCIT Online completion (see Multimedia Appendix 1). Only independent variables or categories of dummy-coded variables were included in regression analyses if they were statistically significantly correlated with outcome variables. Logistic regression was used to examine the extent that the independent variables (ie, child age, child gender, child race, caregiver education, family income, and enrollment in Pocket PCIT Online during COVID-19) predicted the outcome variables (any Pocket PCIT Online engagement and completion; coded 1=yes, 0=no). Two logistic regression analyses were conducted to examine factors potentially associated with any engagement in Pocket PCIT Online (ie, completion of a CDI or PDI homework assignment) and completion of PCIT. All independent (ie, predictor) variables were entered simultaneously into one model or block of the logistic regression model.

For adoption, preliminary correlations were also conducted among independent variables of interest, and only variables with significant correlations with CDI and PDI homework completion rates were entered into linear regression analysis models (see Multimedia Appendix 1). Specifically, linear regression analyses were conducted to examine the extent to which the independent variables (categorical variables dummy coded for the analysis; ie, caregiver education, household income, pre–PSI-SF Parental Distress, and pre–Family Conflict) predicted the adoption variables (CDI homework completion rate and PDI homework completion rate). All predictor variables were entered simultaneously into one model or block of the linear regression model.

Descriptive statistics are provided for caregiver attitudes regarding Pocket PCIT Online, and descriptive information is provided regarding the Pocket PCIT Online website and YouTube metrics.

For implementation, descriptive statistics are provided for caregiver homework completion over the 4-week trial. Finally, the cost per caregiver was calculated for all participants, study completers, and clinical responders by dividing the total website implementation cost by the number of caregivers in each group.

## Results

### Reach

In examining the study sample population (Table 1) relative to the general US population of caregivers of children ages 2‐7 years old descriptively, several differences emerged. Educational attainment in the sample was markedly higher than national averages, with 76.9% (949/1234) of caregivers holding a bachelor’s degree or higher, compared to approximately 34.8% in the general US adult population. This finding was mirrored in household income, where 65.2% (805/1234) of the sample reports earnings at or above the national median (ie, US $74,580 US median income) [65-67]. Racial and ethnic composition of the sample also differed from national demographics, with 66.9% (825/1234) of the sample identifying as non-Hispanic White, and Hispanic representation at 18.2% (225/1234), slightly below national averages. On average, caregivers who enrolled in Pocket PCIT Online reported clinically significant levels of child frequency of positive behaviors (fewer positive behaviors than typical children) and typical levels of parental distress (see Table 1).

### Access

In total, 1480 caregivers initially accessed Pocket PCIT Online. However, 246 (16.6%) caregivers did not complete preintervention measures and were subsequently lost to follow-up. Therefore, sociodemographic characteristics could not be calculated.

### Effectiveness

Significant improvements (2-tailed *t* tests) were observed across all measures from pre- to postintervention (see Table 2). Caregiver reports of the frequency that their child demonstrated positive behaviors (WACB-P Frequency) showed a substantial increase from pre– to post–Pocket PCIT Online completion, *t*_203_=12.49, *P*<.001, *d*=0.87. Statistically significant reductions from pre- to postintervention completion were also observed in the number of behaviors caregivers wanted to change in their child, *t*_203_=−6.01, *P*<.001, *d*=−0.42; parental distress, *t*_203_=−4.27, *P*<.001, *d*=−0.3; and caregiver-reported family conflict, *t*_203_=−4.91, *P*<.001, *d*=−0.35.

**Table 2.** Pre-post changes in Pocket PCIT^a^ Online outcome variables.

Scale	n	Preintervention, mean (SD)	Postintervention, mean (SD)	2-tailed *t* test (*df*)	*P* value	Cohen *d*
WACB-P^b^ Frequency	204	33.33 (6.24)	38.19 (6.64)	12.49 (203)	<.001	0.87
# of Behaviors to Change in Their Child	204	5.17 (2.2)	4.21 (2.31)	–6.01 (203)	<.001	–0.42
PSI-SF^c^ Parental Distress	203	32.48 (8.65)	30.42 (8.74)	–4.27 (203)	<.001	–0.30
Family Conflict	202	2.85 (0.92)	2.60 (0.82)	–4.91 (203)	<.001	–0.35

a PCIT: Parent-Child Interaction Therapy.

b WACB-P: Weekly Assessment of Child Behavior – Positive.

c PSI-SF: Parenting Stress Index – Short Form.

Several linear regression analyses were conducted to determine if family factors impacted the extent to which Pocket PCIT Online impacted child frequency of positive behavior, parental distress, and family conflict, after controlling for preintervention levels (see Table 3). Results from regression analyses indicate that after controlling for preintervention levels, few caregiver factors or sociodemographic characteristics significantly predicted postintervention outcomes. For the WACB-P (frequency of positive child behaviors) outcome, preintervention parental distress was the only significant predictor (β=.49, *P*<.001), suggesting that higher initial parental distress predicted more frequent positive child behaviors at postintervention, even after accounting for preintervention positive behavior levels. This model explained a substantial portion of variance (*R*² change=0.54, *P*<.001).

**Table 3.** Family factors and characteristics that predict family outcomes.^a^

Outcome variables	Predictor variables									
	Pre–WACB-P^b^	Pre–Parental Distress	Pre–Family Conflict	Child race: White	Child race: Black	Child age	Caregiver education: bachelor’s or higher	CDI^c^ total homework completed	*R*^2^ change	*P* value
Post–WACB-P	—^d^	0.49	0.04	−0.05	—	—	0.05	0.10	0.54	<.001
Post–PSI-SF^e^ Parental Distress	0.19	—	−0.18	0.09	—	—	—	—	0.09	.10
Post–Family Conflict	0.01	0.04	—	0.13	0.12	0.07	—	—	0.09	.17

a The first column is the outcome variable that is being predicted. Within each regression analysis, prelevels of the postmeasure were held constant to examine the extent to which additional caregiver factors and sociodemographic characteristics predicted family outcomes. Some predictors are not reported (as indicated by dashes) as they are the prelevels of the outcome variable and because they were not significantly correlated with the outcome variables. Therefore, they were not included in the final multivariate regression models to ensure model stability and in the interest of parsimony. Categorical variables were dummy coded.

b WACB-P: Weekly Assessment of Child Behavior – Positive.

c CDI: Child-Directed Interaction.

d Not applicable.

e PSI-SF: Parenting Stress Index – Short Form.

For parental distress and family conflict outcomes, none of the examined predictors significantly contributed to outcome variance beyond preintervention levels, as evidenced by nonsignificant *R*² change values (*P*=.1 and *P*=.17, respectively). This suggests that initial levels of parental distress and family conflict were the strongest predictors of postintervention outcomes, with minimal contribution from the caregiver factors examined.

Finally, caregivers reported varying degrees of clinical change in their children’s frequency of disruptive behaviors over 1 month. The largest proportion of caregivers (85/204, 41.7%) reported no changes in their children’s disruptive behaviors. However, 35.8% (73/204) of caregivers reported clinically significant improvements in their children’s frequency of positive behaviors. Additionally, 21.6% (44/204) indicated that their children’s frequency of positive behaviors remained within normal limits. Only 1% (2/204) of caregivers reported a clinically significant decrease in child frequency of positive behaviors.

### Adoption

#### Any Engagement and Completion

Among caregivers who completed preintervention measures, 31.8% (393/1234) reported engaging in CDI or PDI homework during the 4-week Pocket PCIT Online intervention, and 16.5% (204/1234) of caregivers completed the intervention. Caregivers completed Pocket PCIT Online at a significantly higher rate (*χ*²_1_=7.98, *P*=.005) prior to the COVID-19 national emergency (21/74, 28.4%) than during or after the onset of COVID-19 (183/1158, 15.8%).

#### Engagement in Pocket PCIT Online

A logistic regression model was used to explore predictors of engagement in Pocket PCIT Online, including child age, child gender, and caregiver education. Only independent variables that correlated with engagement were included in the model. The overall model was not statistically significant (McFadden *R*²=0.16, *P*=.33), indicating that these factors, collectively, did not reliably distinguish between those who engaged in Pocket PCIT Online and those who did not. Examining individual predictors, neither child age (odds ratio [OR] 0.56, 95% CI 0.5-3.2; *P*=.51) nor child gender (female: OR 0.74, 95% CI 0.23-2.37; *P*=.62) was related to engagement. Caregiver education level (bachelor’s degree or higher) approached statistical significance (OR 1, 95% CI 0.99-1; *P*=.06).

A second logistic regression model was used to examine potential predictors of PCIT completion, including child race (American Indian or Alaska Native), household income (above the US median), and enrollment during the COVID-19 pandemic. This model reached statistical significance (*R*²=0.27, *P*=.04), explaining a significant proportion of the variance in PCIT completion. However, none of the predictor variables independently predicted Pocket PCIT completion, including being enrolled in Pocket PCIT Online during COVID-19 (OR 0.38, 95% CI 0.29-1.18; *P*=.13), child race (American Indian or Alaska Native: OR 0.74, 95% CI 0.4-1.36; *P*=.33), or family income level (above median: OR 0.995, 95% CI 0.992-0.998; *P*=.19).

#### CDI and PDI Homework Engagement

A linear regression model was used to examine potential predictors of the rate of CDI homework completion in the Pocket PCIT Online program. The model included preintervention family conflict and child race (White and Asian categories). The overall model was not statistically significant (*R*²=0.07, *P*=.2), and none of the individual predictors were statistically significant (ie, pretreatment family conflict [β=.16], child race White [β=.18], and child race Asian [β=.03]) indicating that the included variables did not reliably predict CDI homework completion rates.

A separate linear regression model was used to predict PDI homework completion. This model included preintervention parental distress, pretreatment family conflict, caregiver education (bachelor’s degree or higher), and household income relative to the US median. Like the CDI model, this overall model for PDI homework completion was not statistically significant (*R*²=0.06, *P*=.42). In this model, the individual predictor effects of preintervention parental distress (β=.08), preintervention family conflict (β=.09), caregiver education at the bachelor’s degree level or higher (β=−.07), and household income above the US median (β=.04) were not statistically significant.

#### Pocket PCIT Caregiver Attitudes

Caregiver attitudes toward the Pocket PCIT Online intervention, as measured by the TAI, were generally positive across multiple domains (see Table 4).

**Table 4.** Pocket PCIT Online caregiver attitudes.

Caregiver perceptions of Pocket PCIT Online	n	Min	Max	Mean (SD)
Regarding techniques of disciplining, I feel I have learned (1=nothing to 5=very many useful techniques)	201	1	5	3.43 (1.06)
Regarding techniques for teaching my child new skills, I feel I have learned (1=nothing to 5=very many useful techniques)	199	1	5	3.45 (1.02)
Regarding the relationship between myself and my child, I feel we get along (1=much worse than before to 5=very much better than before)	201	1	5	3.90 (0.70)
Regarding my confidence in my ability to discipline my child, I feel (1=much less confident to 5=much more confident)	201	1	5	3.72 (0.72)
The major behavior problems that my child presented at home before the Pocket PCIT Online program started are at this time (1=considerably worse to 5=greatly improved)	201	2	5	3.79 (0.60)
I feel that my child’s compliance to my commands or requests is at this time (1=considerably worse to 5=greatly improved)	201	2	5	3.72 (0.64)
Regarding the progress my child has made in his/her general behavior, I am (1=very dissatisfied to 5=very satisfied)	201	2	5	3.84 (0.77)
To what degree has Pocket PCIT Online helped with other general personal or family problems not directly related to the program (1=hindered much more than it helped to 5=helped very much)	201	1	5	3.79 (0.76)
I feel the type of program (Pocket PCIT Online) that was used to help me improve the behaviors of my child was (1=very poor to 5=very good)	201	1	5	3.96 (0.90)
My general feeling about the program (Pocket PCIT Online) I participated in is (1=I disliked it very much to 5=I liked it very much)	201	1	5	4.36 (0.81)

a PCIT: Parent-Child Interaction Therapy.

#### Pocket PCIT Web Page and YouTube Metrics

Given that the web platform could not measure individual user website usage, overall Pocket PCIT page views and Pocket PCIT YouTube metrics are reported as a general estimate of overall engagement over the study period (June 1, 2019, to June 30, 2024). Pages within the website [69] were viewed a total of 32,063 times during the study period. Within Pocket PCIT Online, there were 93 instructional and demonstration videos related to CDI and PDI. Overall, videos were viewed a total of 121,777 times for a total viewing time of 3694.88 hours. This indicates that while only 204 caregivers formally completed Pocket PCIT postintervention measures, it is likely that a significantly higher rate of user engagement of the web platform was not captured via pre-post measure collection.

### Implementation

#### CDI and PDI Homework Completion

Homework completion rates (n=174) revealed varying levels of engagement across the 4-week intervention period (28 days). For CDI homework, caregivers completed tasks on average 50.11% of the days (mean 14.03, SD 6.91 days). PDI homework showed lower completion rates, with caregivers engaging on average 32.07% of the days (mean 8.98, SD 8.50 days). The higher mean and lower SD for CDI homework suggest more consistent completion compared to PDI homework. These findings may reflect the sequential nature of how the Pocket PCIT Online program is presented to users, with CDI skills presented first, serving as a foundation for later PDI skill development, which is presented in later sections of the website.

#### Website Implementation Costs

While the initial development of an mHealth BPT can be costly, the relative cost of maintaining a web-based platform is relatively low. Website hosting and domain hosting costs during the study period cost a total of US $1895. If only study completers (n=204) were included in the cost per participant, the cost of web hosting per participant would only be US $9.29 per caregiver. The costs become much lower (US $1.28 per caregiver) when considering the total number of participants who accessed Pocket PCIT Online (N=1480). Moreover, given that 35.8% (73/204) of study completers reported that their child reported clinically significant improvements in their disruptive behavior, that means that it took only US $26.32 for this intervention to significantly improve one child’s disruptive behavior. That estimate is also assuming that none of the study noncompleters experienced clinically significant improvements in their disruptive behavior and is therefore likely a conservative overestimate of the cost to clinically improve one child’s disruptive behavior.

## Discussion

### Reach, Effectiveness, Adoption, and Implementation of Pocket PCIT Online

This RE-AIM trial evaluated Pocket PCIT Online, a self-directed web-based adaptation of PCIT. This study extends prior mHealth BPT research by providing insights into the reach, effectiveness, adoption, and implementation of this mHealth BPT program. By examining the results through this comprehensive framework, researchers and developers can gain valuable knowledge that can be applied to enhance the accessibility, impact, uptake, and successful execution of other mHealth BPT programs.

### Reach

The sociodemographic characteristics of our sample reveal significant disparities in the reach of Pocket PCIT Online, with an overrepresentation of highly educated, high-income, non-Hispanic White caregivers. These sociodemographic characteristics of the caregivers represented in the study limit the generalizability of our findings and highlight the need for targeted strategies to enhance the intervention’s accessibility for under-resourced and historically underserved populations. Because it was not known if or how Pocket PCIT Online would work, the investigators were conservative with their recruitment approaches (eg, national parenting website and families on a waitlist for actual PCIT services). To address these disparities, we propose several approaches. First, partnering with community organizations and health care providers in underserved areas could improve recruitment of historically underserved communities [70]. Further, advertising this parenting resource beyond just one website may significantly increase who accesses Pocket PCIT Online, including advertising within pediatrician offices, childcare settings, federally funded nutrition programs, faith-based organizations, and social media parenting groups [70]. By enhancing reach to underserved populations, Pocket PCIT Online could potentially more effectively serve as a public health intervention.

### Effectiveness

This 4-week open trial of Pocket PCIT Online was associated with improvements across all measured family outcomes. While the lack of a control group limits causal inferences, these preliminary findings suggest that the intervention may hold promise for families of children with disruptive behaviors. The observed improvements align with previous mHealth BPT research [32,33,35,38], and the magnitude of change in child frequency of positive behaviors exceeds what might be expected from natural recovery alone within 1 month [71]. Of note, approximately 35.8% (73/204) of caregivers reported clinically significant reductions in their children’s disruptive behaviors. These findings are encouraging for Pocket PCIT Online as a potential scalable public health intervention because of the low cost and limited human support required to maintain the intervention over time. While Pocket PCIT Online shows promising initial effectiveness, enhancements in reach, adoption, and implementation are needed to strengthen its potential to broadly impact child behavioral health.

### Adoption and Implementation

Caregiver adoption of Pocket PCIT Online presents both encouraging aspects and significant challenges. Engagement in the intervention was relatively low. Interestingly, no sociodemographic or family factors predicted engagement, CDI or PDI homework completion, or study retention. The absence of significant predictors for adoption is somewhat encouraging, as it suggests that the foundational elements of Pocket PCIT Online are broadly applicable and manageable for caregivers. However, the impact of other important aspects of engagement—including relationships (eg, no human support), expectancy (eg, Pocket PCIT will be helpful), and clarity (eg, understanding of PCIT approach and caregiver skill implementation)—was not measured in this study [72].

Perhaps the most striking challenge of Pocket PCIT Online is the high attrition rate, with only 16.5% (204/1234) of caregivers completing the full intervention. Further, the retention rate was lower during and after the onset of COVID-19. This low retention rate is concerning and warrants further investigation into the factors contributing to discontinuing the intervention. The discrepancy between formal completion rates and overall engagement metrics (page views and video watch time) indicates that many caregivers may be interacting with the content without completing all formal assessments. This highlights a limitation in our ability to fully capture user engagement and suggests that the intervention may have a broader reach than initially apparent from completion rates alone. Future iterations might consider incorporating more robust user tracking and reminders [34], providing incentives for assessment completion, and offering strategies (eg, asynchronous health coach, gamification of intervention features, and peer support forums) that have been shown to increase mHealth intervention engagement [41,73-75].

Despite engagement and retention challenges, caregiver attitudes toward Pocket PCIT Online were generally positive. Participants who completed Pocket PCIT Online reported high satisfaction, perceived improvements in child behavior and the parent-child relationship, and increased confidence in parenting skills. These positive attitudes are encouraging and suggest that Pocket PCIT Online is generally well-received and perceived as beneficial.

As it relates to implementation, the total web hosting cost for families who reported clinically significant improvements (73/204, 35.8%) in their child’s disruptive behavior was only US $26.32 per family. This low cost is notable, given that even the most affordable child psychotherapy sessions in the United States cost approximately US $65 a session, with more typical costs ranging from US $100 to $200 per session [76]. So, Pocket PCIT Online costs the equivalent of about 40% of the cheapest single session of psychotherapy a family could find to affect clinically significant change. Pocket PCIT Online’s potential to deliver clinically significant improvements in child behavior at a fraction of the cost of traditional therapy suggests a promising avenue for expanding access to evidence-based parenting interventions.

### Conclusions

This open trial of Pocket PCIT Online demonstrates preliminary support for the potential of self-directed, mHealth BPT as a scalable public health tool for addressing child disruptive behaviors. The intervention’s effectiveness in improving child behavior and parenting outcomes, coupled with its low implementation costs, highlights its promise for increasing access to evidence-based parenting strategies. However, this open trial also underscores significant challenges in reach, engagement, and retention that must be addressed to maximize the intervention’s impact.

While this open trial of Pocket PCIT Online presents a cost-effective and potentially highly accessible approach to BPT, there are multiple limitations. First, while the study was advertised on a national parenting website and free for use, the resulting sample does not reflect the US national demographics in terms of educational attainment, socioeconomic status, or racial and ethnic diversity per US Census data. Second, like other in-person [24] and mHealth BPT programs [40], limited engagement and high levels of attrition were notable within our sample. The use of self-reports of homework completion as a proxy measure of adoption and implementation limited the extent that other important features of caregiver engagement could be evaluated. Effective measurement of digital intervention engagement requires capturing the multidimensional nature of engagement (physical, affective, and cognitive energies), contextual influences (eg, time of day and response to in-the-moment child behavior problem) shaping responses, the translation process from caregiver motivation to behavior (eg, using PRIDE [Praise, Reflection, Imitation, Describe, and Enjoy] skills with child), and the dynamic, fluctuating patterns through real-time assessment techniques rather than relying solely on retrospective self-report measures [77]. Additions to the Pocket PCIT Online platform (eg, on-demand help chatbot, presentation as interactive learning modules, and audio reading companions) may reduce barriers to engagement. Future research should focus on identifying and addressing barriers to engagement (eg, duration of usage, number of times modules reviewed, number of modules completed, and screen readers for those with reading difficulties) and exploring ways to better capture and encourage sustained user participation in mHealth BPT interventions [73]. While web page and YouTube metrics are helpful for understanding broader caregiver use of Pocket PCIT Online, this approach did not allow us to examine individual user metrics. Shifting the website content to an online learning management system that includes individual course progression and duration of use would provide far more useful quantitative measures of individual caregiver engagement with Pocket PCIT Online content. Third, due to both attrition and the lack of included follow-up measures due to resource constraints, the *maintenance* aspect of RE-AIM was unable to be evaluated in this study. As such, long-term changes in family outcomes could not be evaluated. Finally, the open trial did not include a comparison group and did not ask families if they were concurrently receiving other related services. Future research should include randomized controlled trials comparing Pocket PCIT Online with and without human support (ie, coaching and skill feedback). Ultimately, such research and intervention refinement efforts may contribute to reducing the prevalence and long-term consequences of childhood disruptive behaviors on a population level.

## Supplementary material

10.2196/69887 Multimedia Appendix 1 Correlations among all study variables of interest.

## Abbreviations

BPT: behavioral parent training
CDI: Child-Directed Interaction
FIML: full information maximum likelihood
mHealth: mobile health
MLR: maximum likelihood estimation
OR: odds ratio
PCIT: Parent-Child Interaction Therapy
PDI: Parent-Directed Interaction
PRIDE: Praise, Reflection, Imitation, Describe, and Enjoy
PSI-SF: Parenting Stress Index – Short Form
PSI-SF-4: Parenting Stress Index – Short Form, Fourth Edition
RE-AIM: Reach, Effectiveness, Adoption, Implementation, and Maintenance
REDCap: Research Electronic Data Capture
TAI: Therapy Attitude Inventory
WACB-P: Weekly Assessment of Child Behavior – Positive
