# Supplementary material for: Pocket Parent-Child Interaction Therapy (PCIT) Online for Young Children With Disruptive Behaviors: Open Trial
Source: J Med Internet Res. 2025 Aug 8;27:e69887. doi: 10.2196/69887 (PMC12334141; doi:10.2196/69887)
Supplement: Multimedia Appendix 1 [file jmir-v27-e69887-s001.docx]

# Correlations Among All Study Variables of Interest

| **Variable** | **1** | **2** | **3** | **4** | **5** | **6** | **7** | **8** | **9** | **10** | **11** | **12** | **13** | **14** | **15** | **16** | **17** | **18** | **19** | **20** | **21** | **22** |
| --- | --- | --- | --- | --- | --- | --- | --- | --- | --- | --- | --- | --- | --- | --- | --- | --- | --- | --- | --- | --- | --- | --- |
| 1. WACB-P Raw Score (Post) | — |  |  |  |  |  |  |  |  |  |  |  |  |  |  |  |  |  |  |  |  |  |
| 2. Post Family Conflict | -.22** | — |  |  |  |  |  |  |  |  |  |  |  |  |  |  |  |  |  |  |  |  |
| 3. Post PSI Parental Distress | -.22** | .33** | — |  |  |  |  |  |  |  |  |  |  |  |  |  |  |  |  |  |  |  |
| 4. CDI Total Homework | .21* | -.16 | -.06 | — |  |  |  |  |  |  |  |  |  |  |  |  |  |  |  |  |  |  |
| 5. PDI Total Homework | .05 | .06 | .04 | .04 | — |  |  |  |  |  |  |  |  |  |  |  |  |  |  |  |  |  |
| 6. Completed Pocket PCIT | — | — | — | .06 | .04 | — |  |  |  |  |  |  |  |  |  |  |  |  |  |  |  |  |
| 7. Any Pocket PCIT Engagement | .06 | .08 | .00 | .31** | .16* | .58** | — |  |  |  |  |  |  |  |  |  |  |  |  |  |  |  |
| 8. Pre-WACB-P Raw Score | .63** | -.16* | -.27** | -.08 | .04 | .05 | .05 | — |  |  |  |  |  |  |  |  |  |  |  |  |  |  |
| 9. Pre-Family Conflict | -.16* | .65** | .32** | -.21** | -.27** | -.01 | .02 | -.21** | — |  |  |  |  |  |  |  |  |  |  |  |  |  |
| 10. Pre-PSI-SF Parental Distress | -.16* | .30** | .69** | -.06 | -.21** | -.04 | -.02 | -.30** | .35** | — |  |  |  |  |  |  |  |  |  |  |  |  |
| 11. Child: American Indian/Alaska Native | — | — | — | -.01 | .10 | -.06* | .01 | -.04 | .04 | .03 | — |  |  |  |  |  |  |  |  |  |  |  |
| 12. Child: Asian | .19** | .05 | -.18** | -.17* | -.12 | .01 | -.01 | .15** | .02 | .02 | -.04 | — |  |  |  |  |  |  |  |  |  |  |
| 13. Child: Black/African American | .03 | -.24** | -.12 | .02 | .10 | .04 | .05 | -.02 | .00 | -.01 | -.04 | -.08** | — |  |  |  |  |  |  |  |  |  |
| 14. Child: Native Hawaiian/Pacific Islander | .01 | -.04 | .04 | -.13 | -.03 | .00 | -.04 | .00 | .00 | .02 | -.01 | -.03 | -.03 | — |  |  |  |  |  |  |  |  |
| 15. Child: White | -.16* | .15* | .21** | .15* | .00 | -.01 | -.02 | -.08** | -.03 | -.03 | -.30** | -.64** | -.58** | -.22** | — |  |  |  |  |  |  |  |
| 16. Child: Hispanic/Latino | -.14 | -.08 | -.05 | .12 | .08 | .00 | .00 | -.04 | -.09** | -.07* | .09** | -.09** | -.03 | .03 | .04 | — |  |  |  |  |  |  |
| 17. Child: Not Hispanic/Latino | .14 | .08 | .05 | -.12 | -.08 | .00 | .00 | .04 | .09** | .07* | -.09** | .09** | .03 | -.03 | -.04 | -1.00** | — |  |  |  |  |  |
| 18. US National Median Household Income | .04 | -.08 | -.04 | .00 | -.20** | .06* | .05 | .17** | -.03 | -.08** | -.12** | -.05 | -.04 | -.04 | .11** | -.10** | .10** | — |  |  |  |  |
| 19. Education Level- Bachelor’s Degree | .14* | .04 | -.04 | -.14 | -.24** | .05 | .07* | .21** | -.02 | -.05 | -.09** | .07* | -.03 | .00 | .00 | -.05 | -.05 | .46** | — |  |  |  |
| 20. Child Age | .12 | .21** | .04 | -.05 | .04 | -.04 | -.06* | .23** | .11** | .02 | .02 | -.02 | .03 | -.02 | -.12** | .12** | -.03 | .02 | .03 | — |  |  |
| 21. Child Gender | .10 | -.06 | -.00 | .08 | -.08 | -.04 | -.06* | .13** | .03 | -.00 | -.01 | -.02 | -.01 | -.02 | .02 | .01 | -.01 | -.02 | .02 | .02 | — |  |
| 22. Enrolled during COVID-19 | .13 | .03 | .05 | -.08 | -.05 | -.0908** | -.056* | .05 | .04 | -.01 | .04 | -.04 | -.01 | .03 | .02 | -.12 | .12* | .08** | -.01 | .05 | .03 | — |

*Note.* N ranges from 142 to 1249 on correlations due to missing data or participant being lost to follow up. WACB-P= Weekly Assessment of Child Behavior- Positive PSI = Parenting Stress Index; CDI = Child Directed Interaction; PDI = Parent Directed Interaction; PCIT = Parent-Child Interaction Therapy; US National Median Household Income: 1= above US national household median income, 0= at or below US national median household income; Education Level: 1= Bachelor’s Degree or higher, 0= below Bachelor’s Degree; Child Gender: 1= male, 2= female. **P* < .05. ** P < .01. Dashes (—) indicate that the correlation could not be computed due to at least one of the variables being constant or all values for one variable being missing.
